# Protective effects of garlic aquous extract (*Allium sativum*), vitamin E, and N-acetylcysteine on reproductive quality of male rats exposed to lead

**Published:** 2013

**Authors:** Reza Asadpour, Mehdi Azari, Marzie Hejazi, Hossein Tayefi, Neda Zaboli

**Affiliations:** 1 *Department of Clinical Science, Faculty of Veterinary Medicine, University of Tabriz, Tabriz, Iran; *; 2 *Department of Basic science, Faculty of Veterinary Medicine, University of Tabriz, Tabriz, Iran.*

**Keywords:** Garlic extract, Lead, N-acetylcysteine, Rat, Vitamin E

## Abstract

The objective of present study was to investigate the effects of aqueous garlic extracts, vitamin E and N-acetylcysteine on lead-induced lipid peroxidation, changes in antioxidant defense system and semen quality in the rat testes. Twenty-five male rats were divided into five groups. Animals within different treatment groups were maintained on their respective diets for 35 days as follows: group 1 rats served as control and received water and standard pellets as food *ad libitum*; group 2 received lead acetate by gavage (1000 ppm); group 3 was treated with *A. sativum* extract (400 mg kg^-1^, by gavage) plus lead acetate (1000 ppm); group 4 was treated orally with vitamin E (300 mg of alpha-tocopherol per kg of chow) plus lead acetate (1000 ppm); group 5 was treated orally with N-acetylcysteine (800 ppm)plus lead acetate (1000 ppm). The weights of testes, epididymis, epididymal sperm count, viable and motile sperms decreased significantly (*p *< 0.05) in lead-exposed rats. However treatment with vitamin E and aqueous garlic extract resulted in a significant (*p *< 0.05) increase in sperm motility and viability. Exposure to lead acetate significantly increased malondialdehyde (MDA) level with a significant decrease in the superoxide dismutase (SOD) activities in the testes of rats while co-administration of vitamin E and lead caused a significant (*p *< 0.05) decrease in MDA concentration compared with lead-exposed group. These results suggest that both vitamin E and in to a lesser extent aqueous garlic extract have a potent antioxidant protection in the testes of rat against the lead-induced oxidative stress.

## Introduction

Lead (Pb) is one of the ubiquitous non-essential heavy metals in the environment. There are evidences showing that lead toxicity has many undesired effects, including neurological, behavioral, immunological, renal, hepatic, male reproductive dysfunctions.^1^^-^^6^ From the mechanisms by which lead can damage the cells of different organs, production of reactive oxygen substances (ROS) are important and they have major impact in development of oxidative stress.^7^ In general, to overcome the ROS attack, animal cells are equipped with an intrinsic scavenging system with enzymes and anti-oxidant molecules.^7^ Thus, a failure in the neutralizing system against ROS adversely affects the organs and their functions. Recently, it has been reported that alterations in the activity levels of antioxidant enzymes and increase in the lipid peroxidation products causes hepatotoxicity and nephrotoxicity in lead exposed rats.^8^ Sainath *et al.* demonstrated that exposure to lead acetate significantly increased malondialdehyde levels with a significant decrease in superoxide dismutase and catalase (CAT) activities in testes of rats.^9^ It seems that lead toxicity, at least in part, originates from oxidative stress and thus, a therapeutic strategy to increase the antioxidant capacity of cells against lead poisoning is considered important. This may be accomplished by either removal of lead from the tissues or prevention of its interactions with cellular macro-molecules and by provoking the cellular antioxidant defenses through endogenous supplementation of anti-oxidant molecules. Among those strategies, chelating therapy is widely used to treat lead poisoning. The chelating agents used to prevent lead accumulation in the soft tissues include vitamins,^10^ thiol compounds which known to displace toxic metals from the tissues and the modulation of various biochemical aspects. 

Vitamin E is the primary liposoluble antioxidant, which may have an important role in scavenging free oxygen radicals and in stabilizing cell membranes maintaining its integrity.^11^ In lead-exposed rats, supplementation of vitamin E and/or C reduced sperm ROS generation, preventing loss of sperm motility and oocyte penetration capacity.^10^

N-acetylcysteine (NAC), the thiol based antioxidant, plays an important role both in the protection of cellular constituents against oxidative damage and in the detoxification of many electrophiles. The hypothetical action of NAC originates from its ability to stimulate and to sustain the intracellular levels of reduced glutathione levels and to detoxify ROS^12^. The other advantage of NAC is its efficiency to chelate toxic metals.^12^

In this respect, a special attention was paid to garlic (*Allium sativum*). This plant is used for a long time both in cooking and as a medicinal plant.^13^ Extensive studies have been carried out on garlic (*Allium sativum L*.) have reported the presence of two main classes of antioxidant components, namely flavonoids and sulfur-containing compounds (diallyl sulfide, trisulfide and allylcysteine).^14^^,^^15^ These are likely to play an important role in the widely demonstrated biological effects of garlic, which include antitumor, hypolipidemic, hypocholesterolemic, antiatherosclerotic, antioxidant, and immunomodulatory effects.^16^^-^^21^ However, the protective effects of garlic extract, vitamin E and NAC on male reproduction have not been studied. Thus the objective of the present study was to investigate the potential protective properties of *A. sativum* aqueous extracts, vitamin E and NAC against lipid peroxidation, and changes in enzymatic antioxidant defense system induced by lead in the rat testes.

## Materials and Methods

**Chemicals.** Lead citrate, D- and L- alpha-tocopherol (Vitamin E), and N-acetylcysteine, were purchased from Sigma (Sigma Chemical Co., St. Louis, USA). All other chemicals used were of analytical grade and obtained from Merck-Schuchardt (Munich, Germany).

**Plant material. **Fresh garlic was collected in August 2010 from University garden, Tabriz, Iran. It was indentified and authenticated by a quilified botanist in the herbarium of faculty of agriculture, University of Tabriz (Tabriz, Iran).

**Preparation of aqueous garlic extract (AGE).** Aqueous garlic extract was prepared using fresh garlic by modified method of Martha *et al*.^12^ Thirty gram of garlic was homogenized in 100 mL of cold distilled water. The homogenized mixture was filtered three times through cheese cloth. The mixture was centrifuged at 200 *g* for 10 min and the clear supernatant was collected. The concentration of this garlic preparation was considered to be 500 mg mL^-1^, on the basis of the weight of the starting material (30 g per 100 mL). Fresh extract was prepared each day before administration.^12^

**Animals.** Twenty-five healthy adult male Wistar rats weighing 210 ± 10 g were used. The animals were obtained from animal house of University of Tabriz. The animals were housed in cages at 23 ± 1 ˚C and exposed to 12-12 hr light-dark cycle. They had access to a standard rodent laboratory diet and drinking water *ad libitum* throughout the study.

**Experimental design.** After one week of acclimatization period, the rats were divided into five groups of five rats each. Animals within different treatment groups were maintained on their respective diets for 35 days as follows: group 1, rats served as control (C) and received water and standard pellets as food *ad libitum*; group 2, received lead acetate by gavage (1000 ppm); group 3 was treated with *A. sativum* extract (400 mg kg^-1^, by gavage) plus lead acetate (1000 ppm); group 4 was treated orally with vitamin E (300 mg of alpha tocopherol per kg of chow) plus lead acetate (1000 ppm); group5 was treated orally N-acetyl-cysteine (800 ppm) plus lead acetate (1000 ppm).^22^^-^^24^

**Collection of sample. **At the end of treatment period, final body weight of each animal was recorded and the animals were euthanized by using overdose of anesthetic ether.^9^ Blood was taken by cardiac puncture and was allowed to clot at room temperature and was centrifuged at 2000 rpm for 10 min to separate serum. The serum samples were stored at -20 ˚C in microfuge tubes and were used for analyzing the level of hormones. 

**Tissue somatic index (TSI).** At the end of treatment period, and after blood samples were collected, all animals were killed euthanized. Afterward, the testes and accessory sex organs were dissected .They were weighed to the nearest milligram on an electronic balance (Model: BL-220H, Shimadzu, Tokyo, Japan). The tissue indices were calculated using the following formula:

$\mathrm{Tissue somatic index}=\frac{\mathrm{Weight of the tissue}(g)}{\mathrm{Weight of the body}(g)}\times 100$

**Sperm analysis. **Epididymal sperm count and sperm progressive motility were evaluated by the method of Linder *et al.*^24^ Accordingly, epididymal spermatozoa were obtained by mincing the epididymis with anatomical scissors in 5 mL of Ham’s F12 medium and incubated at 32 ˚C for 2 min. An aliquot of this solution was placed in Neubauer hemocytometer and motile sperm were counted by using microscope at 400 × magnification. Non-motile sperm numbers were first determined, followed by counting of total sperm. Sperm motility was expressed as a percent of motile sperm of the total counted sperm. Percentage of morphologically abnormal spermatozoa was determined by the method described by Evans and Maxwell.^25^ According to this method, slides were prepared with Wells and Awa stains for morphological examination and for estimination of live-dead ratio of sperms a satin containing 1% Eosin B and 5% Nigrosine in 3% Sodium citrate dehydrate solution was prepared for live-dead ratio. A total of 400 sperm cells were counted on each slide under light microscope at 400 × magnification.

**Determination of serum testosterone levels. **Serum level of testosterone was determined by enzyme linked immunosorbant assay (ELISA) using a commercial kits (Diagnostic System Laboratories Inc., Webster, USA). The assay was done strictly according to the procedure given along with the kit. The sensitivity of the assay was calculated as 0.002 ng and intra-assay variation was 5%. All of the samples were run at the same time to avoid inter-aassay variation. The serum level of testosterone was expressed as ng mL^-1^.

The Luteinizing hormone (LH), was measured using the Vidas parametric system. The procedure was as described in the manufacturer’s manual (BioMérieux, St. Louis, USA). All samples for a given experiment were performed within the same assay. Intra- and inter-assay coefficients of variation for the rat assay were 5% and 11%, respectively.

**Tissue preparation.** At the end of treatment period, and after blood samples were collected, all animals were killed and the testis tissue of each animal was dissected, weighed and homogenized in phosphate buffer (pH 7.4) to give a 20% w/v homogenate. This homogenate was centrifuged at 1700 rpm for 10 min at 4 ˚C and the supernatant was stored at -70 ^°^C until analysis. The super-natant was used for antioxidant enzyme activity assays and for total protein determination.^26^

**Biochemical assays. **The total protein content of the homogenized testes was determined by the method of Lowry *et al*.^27^ Catalase activity was measured in the supernatant by the Goth method, 50 µL samples were mixed with 1 mL of 65 mmol mL^-1^ hydrogen peroxide in 60 mmol L^-1^ sodium potassium phosphate buffer (pH 7.4) and incubation was performed at 37 ˚C for 60 sec.^28^ One unit of CAT represents the amount of enzymes that decomposes 1 µmol of hydrogen peroxide per min. The enzymatic reaction was terminated with 1 mL of 32.4 mmol L^-1^ ammonium molybdate, and hydrogen peroxide was measured at 405 nm using a spectrophotometer (Unico UV-2100 PC, Dayton, USA). 

Superoxide dismutase activity was assayed by reduction of nitro blue tetrazolium (NBT) with reduced nicotinamide adenine dinucelotide (NADH) mediated by phenazine methosulfate (PMS) which is inhibited upon addition of SOD.^29^ In a cuvette, 2.6 mL of phosphate buffer (0.017 mol, pH 8.3) was mixed with 0.1 mL each of PMS (0.093 mmol), NBT (1.5 mmol) and supernatant prepared as above at 25 ˚C. After adding 0.1 mL of NADH (2.34 mmol), the reaction was started, and an increase in absorbance was recorded at 560 nm for 3.5 min at 30 sec intervals. A unit of SOD was defined as the activity of enzyme required for suppressing the increase in absorbance by 50%.

Glutathione peroxidase (GPx) activity was measured by the method of Paglia and Valentine.^30^ The assay was based on nicotinamide adenine dinucleotide phosphate (NADPH)-coupled reaction where oxidized glutathione, produced upon reduction of organic peroxide (tertabutyl hydroperoxide) by GPx, was recycled to its reduced state by utilizing enzymes glutathione reductase and NADPH. The oxidation of NADPH to NADP^+^ was associated with a decrease in absorbance at 340 nm, providing a spectrophotometric means for monitoring GPx activity. The rate of decline in absorbance at 340 nm was directly proportional to the GPx activity.

**Statistical analysis. **Data are expressed as mean ± SEM. The data was analyzed using SPSS version 16 (SPSS Inc., Chicago, USA). Statistical analysis was performed using analysis of variance (ANOVA) followed by Tukey test and the level of significance was set at *p* < 0.05.

## Results

The effect of lead on total epididymal sperm count, sperm motility and viability is shown in Table 1. No significant changes in the weights of testes were observed in rats after lead administration. On the other hand, treatment of male rats with lead acetate caused a significant (*p *< 0.05) decrease in sperm motility and viability compared to the control group. Whereas administration of vitamin E to lead exposed animals resulted in statistically significant increase in sperm motility and viability when compared with the lead treated rats (*p *< 0.05).

**Table 1:** Supplementation with aqueous garlic extract (AGE), vitamin E and N-acetylcysteine (NAC) against lead acetate-induced change in sperm parameters in male rat (Mean ± SEM).

**Sperm parameters**	**Control**		**Lead**	**Lead+ AGE**	**Lead + Vitamin E**	**Lead + NAC**
**Tissue somatic index**		0.60 ± 0.04	0.50 ± 0.04	0.61 ± 0.07^b^	0.66 ± 0.03^b^	0.57 ± 0.08
**Epididymal sperm count (10** ^6^ ** per mL)**		33.75 ± 2.10	19.25 ± 1.40^a^	25.20 ± 1.29^a^	24.12 ± 0.78^a^	27.87 ± 1.54^b^
**Sperm motility (%)**		73.00 ± 1.52	62.12 ± 0.66^a^	66.66 ± 0.76^a^	69.83 ± 1.81^b^	64.83 ± 1.24^a^
**Sperm viability (%)**		91.00 ± 1.00	73.00 ± 1.18^a^	82.5 ± 1.50^a^,^b^	80.16 ± 1.32^a^,^b^	78.66 ± 2.44^a^

a indicates statistically significant difference with control group (*p* < 0.05);

b indicates statistically significant difference with Lead group (*p* < 0.05); Mean values with same superscripts in rows do not differ significantly from each other.

Also, treatment with aqueous garlic extract caused a statistically significant increase in sperm viability and minimized the toxic effects of lead acetate (*p* < 0.05). Significant decrease in sperm concentration was recorded in the lead-treated group. Administration of NAC to lead exposed animals resulted in statistically significant increase in sperm concentration when compared with lead exposed group (*p *< 0.05). No significant change in tissue somatic indices was found in rats treated with vitamin E, NAC and aqueous garlic extract when compared to the lead-treated animals.

Effects of lead alone and concurrently with aqueous garlic extract, vitamin E and NAC on antioxidant activity and lipid peroxidation are shown in Table 2. Significant elevation in the activity levels of SOD were observed with remarkably reduced levels of lipid peroxidation products in the lead-exposed rats co-administered with vitamin E compared to the lead-exposed rats (*p *< 0.05).

No significant change in GPx was found in the rats treated with vitamin E, NAC and aqueous garlic extract groups in comparison with the control and lead-exposed groups. Malondialdehyde concentration was significantly increased in the testes of rats after treating with lead while administration of vitamin E simultaneously with lead resulted in a significant (p < 0.05) decrease in MDA concentration when compared with the lead exposed group. No significant changes in testosterone levels were observed in rats after lead administration. However, there was a decrease in serum levels of this hormone in the lead- exposed group. On the other hand, a significant (*p *< 0.05) increase was observed in LH level in the rats exposed to lead plus NAC in comparison with lead plus aqueous garlic extract group. Catalase decreased markedly after the treatment with lead .Garlic extract was unable to increase that but both vitamin E and NAC increased its level significantly. Garlic extract was able to improve sperm parameters and quality better than NAC and it is completely comparable to that of vitamin E in this respect (Table3).

**Table 2:** Supplementation with aqueous garlic extract (AGE), vitamin E and N-acetylcysteine (NAC) against lead acetate- induced change in oxidative stress related parameters (Mean ± SEM).

**Parameters**	**Control**	**Lead**	**Lead+ AGE**	**Lead + Vitamin E**	**Lead + NAC**
**Superoxide dismutase (U mg** ^-1^ **)**	255.29 ± 3.59	232.82 ± 13.72	265.18 ± 3.07	266.98 ± 3.98^b^	269.68 ± 6.88
**Glutathione peroxidase (U mg** ^-1^ **)**	25.88 ± 2.43	19.38 ± 1.84	26.97 ± 2.33	24.19 ± 2.06	28.66 ± 3.82
**Catalase (U mg** ^-1^ **)**	81.95 ± 11.65	43.60 ± 12.84	21.16 ± 6.49^a^	48.50 ± 4.78	41.68 ± 12.85
**Malondialdehyde (µmol mg** ^-1^ **)**	71.46 ± 5.95	96.90 ± 14.26	87.13 ± 6.84	39.46 ± 6.14^b^,c	64.83 ± 15.11
**Total protein (g dL** ^-1^ **)**	19.38 ± 1.84	25.88 ± 2.43	26.97 ± 2.33	24.19 ± 2.06	28.66 ± 3.82

a indicates statistically significant difference with control group (*p* < 0.05);

b indicates statistically significant difference with Lead group (*p* < 0.05);

c indicates statistically significant difference with Lead+ AGE (*p* < 0.05); Mean values with same superscripts in rows do not differ significantly from each other.

**Table 3:** Supplementation with aqueous garlic extract (AGE), vitamin E and N-acetylcysteine (NAC) against lead acetate- induced change in some hormonal variables in rat

**Parameters**	**Control**	**Lead**	**Lead+ AGE**	**Lead + Vitamin E**	**Lead + NAC**
**Testosterone (ng mL** ^-1^ **)**	1.58 ± 0.78	0.83 ± 0.38	1.82 ± 1.26	2.00 ± 1.02	2.90 ± 1.05
**Luteinizing hormone (ng mL** ^-1^ **)**	0.48 ± 0.08	0.14 ± 0.50	0.75 ± 0.04	0.60 ± 0.20	2.12 ± 1.60*

* Asterisk indicates statistically significant difference with Lead + AGE (*p *< 0.05).

## Discussion

The present study addresses the protective role of vitamin E and to a lesser extent aqueous garlic extract on lead-induced testicular damage as evidenced by restoration of weight of testes, reduced levels of testicular lipid peroxidation products with increased activities of SOD and catalase, improvement in sperm quantity and quality and increased testicular steroidogenesis. In the present study, administration of lead caused reduction in epididymal sperm concentration, suppression of sperm progressive motility and live/dead count. A significant decrease in the weight of testes and epididymis was observed in the lead-treated rats. Our results are in agreement with studies of Hamadouche *et al*. who reported that lead acetate at a dose level of 500 mg L^-1^ significantly decreased the weight of testes.^33^ The weight of the testes is also largely dependent on the mass of the differentiated spermatogenic cells and reduction in the testicular weight indicates germ cells loss.^9^

The sperm endpoints such as epididymal sperm count, sperm motility and sperm viability were significantly decreased in the lead-treated rats. It has been shown that lead acetate intoxication during spermatogenesis can delay spermiation as well as release of immature spermatogenic cells in the tubules of testes. ^34^ The results are also in agreement with earlier reports of Hamadouche *et al*. who reported that lead reduces significantly epidimyal and testicular sperm counts including daily sperm production^33^. One possible explanation is that lead can have a direct influence on sperm quality. Another explanation is that H_2_O_2 _as one of the lipid peroxidation products might diffuse across the membrane and affect the vital enzymes in the sperms thereby resulting in decreased sperm motility. We demonstrated that sperm motility and viability in lead and vitamin E group was higher than the lead group. This study is in agree-ment with earlier reports of Hsu *et al*. who demonstrated that lead-exposed rats supplemented with vitamin E in their drinking water resulted in significant increases of sperm motility.^10^ Sperm viability was also significantly increased in the rats subjected to co-administration of garlic extract as compared to lead-exposed rats (*p *< 0.05). These findings indicate the possible role of garlic extract, characterized by increased level of antioxidant enzymes and decreased lipid peroxidation in testis, in combating the testicular toxicity of lead.^19^

Sperm plasma membrane, being rich in poly-unsaturated fatty acids, is highly susceptible to ROS attack. To negate the harmful effects of ROS, testes are equipped with a powerful antioxidant defense system involving enzymes like SOD and catalase.^35^ Superoxide dismutase is considered the first line of defense against deleterious effects of oxygen radicals in the cell by catalyzing the dismutation of superoxide anion radicals to H_2_O_2_, which is readily degraded by catalase. In a biological system, the antioxidant enzymes catalase protects SOD inactivation by H_2_O_2_, while the SOD reciprocally protects catalase against inhibition by superoxide anion. Thus, balance of this enzyme system may be essential to eliminate superoxide and peroxide radicals generated in the tissues. In the present study, exposure to lead decreased the activities of SOD and catalase, and concomitantly increased the levels of lipid peroxidation in the testis. The reduction in activities of antioxidant enzymes shows the failure of primary antioxidant system to act against free radicals. In consistent with our results, Sharma *et al*. reported a reduction of SOD activities in lead exposed rats while Soltaninejad *et al*. found higher activity of SOD.^22^^,^^36^ Vitamin E is a chain breaking antioxidant and supplementation with vitamin E during lead intoxication reduced the levels of lead -induced oxidative damage in the testis. Whereas in the group that received lead and aqueous garlic extract together in catalase activity is evident and it might be due to the ineffective scavenging of H_2_O_2._

Different effects of lead on the activities of GPx were previously noticed. No significant change in GPx was found in rats treated with vitamin E, NAC and aqueous garlic extract groups in comparison with the control and lead-exposed groups. The increased amount of GPx activity in the testis of NAC - treated lead exposed rats indicated the chelating potential of NAC. It is well established that NAC is a very effective precursor and stimulator of glutathione synthesis and in many disorders it has been demonstrated that NAC augments glutathione production.^23^

 In this study, treatment with lead resulted in a significant increase in lipid peroxidation as indicated by the significant increase in MDA content of the testis. Increased lipid peroxidation may indicate an increased generation of ROS, which can cause damage to sperm and other cytoplasmic organelle membrane structures through peroxidation of lipids, proteins and nucleotides, thereby altering sperm motility. The stimulation of lipid per-oxidation observed in the current study as a result of lead exposure could be due to the formation of free radicals through an exhaustion of antioxidants as shown by Soltaninejad *et al*. vitamins E decreased significantly lipid peroxidation as compared to the control rats.^36^ In many studies vitamin E neutralizes lipid peroxidation and unsaturated membrane lipids because of its oxygen scavenging effect.^38^

Testosterone synthesis in Leydig cells and spermatogenesis in seminiferous tubules are the two energy requiring processes in the testis. Testosterone is secreted from Leydig cells acts on Sertoli cells in seminiferous tubules to create an environment with nutritional and hormonal factors in which normal progression of germ cells happens through spermatogenic cycle. Spermatogenesis in mammals is used as an important indicator of the chemical-induced toxicity on male reproduction. The observed reduction in the number of sperm in epididymis and decrease in the weight of reproductive organs may reflect less bio-availability or production of androgen in lead exposed rats. It was established earlier that lead causes testicular toxicity by germ cell degeneration and inhibits androgen production in adult male rats, probably by affecting pituitary luteinizing hormones and thereby inhibiting Leydig cell testosterone production which in turn compromises spermatogenesis.^32^ The decrease in the serum level of testosterone in rats exposed to lead may be due to both direct effect on testis and suppression of luteinizing hormone secretion. There is a significant difference in LH level in the group of animals that received aqueous garlic extract simultaneously with lead. Different protective pathways were proposed to explain the beneficial effect of garlic components. It includes ROS scavenging, inhibition of low density lipoproteins (LDL) oxidation, protection of endothelial cell integrity by inhibition of lipid peroxidation induced injury, inhibition of homocystein ethiolactone formation, improving cellular scavenging enzyme such as superoxide dismutase, catalase, glutathione peroxidase and inhibition of nuclear factor activation.^39^

It could be concluded that co-administration of aqueous extract of garlic and vitamin E, reduces lead-induced oxidative stress by decreasing lipid peroxidation and activating anti-oxidant enzymes in the testes, thereby ameliorating lead induced suppressed reproduction in male rats. 
